# Irrigation, risk aversion, and water right priority under water supply uncertainty

**DOI:** 10.1002/2016WR019779

**Published:** 2017-09-14

**Authors:** Man Li, Wenchao Xu, Mark W. Rosegrant

**Affiliations:** ^1^ Environment and Production Technology Division, International Food Policy Research Institute Washington DC USA; ^2^ Department of Economics School of Economics and WISE, Xiamen University Xiamen China

**Keywords:** water rights, skewness risk, water supply uncertainty, irrigated land allocation, climate change

## Abstract

This paper explores the impacts of a water right's allocative priority—as an indicator of farmers' risk‐bearing ability—on land irrigation under water supply uncertainty. We develop and use an economic model to simulate farmers' land irrigation decision and associated economic returns in eastern Idaho. Results indicate that the optimal acreage of land irrigated increases with water right priority when hydroclimate risk exhibits a negatively skewed or right‐truncated distribution. Simulation results suggest that prior appropriation enables senior water rights holders to allocate a higher proportion of their land to irrigation, 6 times as much as junior rights holders do, creating a gap in the annual expected net revenue reaching up to $141.4 acre^−1^ or $55,800 per farm between the two groups. The optimal irrigated acreage, expected net revenue, and shadow value of a water right's priority are subject to substantial changes under a changing climate in the future, where temporal variation in water supply risks significantly affects the profitability of agricultural land use under the priority‐based water sharing mechanism.

## Key Points

- Develops a theoretical framework to explicitly model the influence of water institution on a farmer's land irrigation decision under water supply uncertainty
- The optimal acreage of land irrigated increases with a water right's priority when water supply uncertainty exhibits a left‐skewed or right‐truncated distribution
- Significant differences exist in agricultural productivity between senior and junior water right holders

## 1 Introduction

Water scarcity is among the top global risks identified by the World Economic Forum and has drawn considerable attention from academic scholars and policymakers [*WEF*, 2015]. In many parts of the world, water supply is highly uncertain and water storage and conveyance facilities are inadequate [*Hanemann*, 2006]. In some places, water resources are poorly managed, leading to widespread inefficient use both physically and economically [*Cai et al*., 2003; *Jensen et al*., 1990; *Schaible and Aillery*, 2012]. Agricultural economists, hydrologists, and institutional governance experts have developed a variety of strategies to improve the efficiency of water use and enhance adaptive capacity of rural‐urban communities [*Harou et al*., 2009; *Rosegrant and Binswanger*, 1994; *Rosegrant et al*., 2017; *Smit and Wandel*, 2006; *Thompson*, 1993; *Wada et al*., 2014, among others]. A subset of agricultural economists has focused on understanding how water scarcity and water supply uncertainty influence individual farmers' irrigation decisions [e.g., *John et al*., 2005; *Kaiser et al*., 1993; *Mejias et al*., 2004; *Peck and Adams*, 2010, 2012, among others]. Yet it remains unclear how institutional water sharing arrangements affect this decision‐making process. This paper presents an economic model to shed light on this issue.

Climatic factors have long been recognized as the primary source of water supply uncertainty. Temperature and precipitation, for example, influence the timing and volume of streamflow in river basins. In the U.S. West, irrigated agriculture relies heavily on snowmelt‐driven streamflow originating from high‐elevation areas. A low‐snow year can reduce the amount of water available for irrigation downstream, and earlier‐than‐normal spring snowmelt can cause streamflow to peak one or more weeks earlier than usual. Both cases can result in abnormally low spring and early summer river discharge [*Cayan et al*., 2001; *Stewart et al*., 2005]. In addition to the above‐mentioned stressors, global climate change is causing more frequent extreme weather events such as excessive heat and persistent drought, which exacerbate water supply uncertainty [*Romero‐Lankao et al*., 2014].

Continuing debate over water markets, particularly among watershed managers and practitioners, highlights the complexity of using water institutions to cope with increasing water supply uncertainty. Though such water institutions are typically operated at a regional level, they can substantially affect individual farmers' responses to risk. For instance, under the riparian doctrine in the U.S. East, each property owner with river frontage has a right to unimpaired use of the waterway. These water users bear a supply risk that is in proportion to their frontage on the water source.

In contrast, under the prior appropriation doctrine of the U.S. West, water users' rights are sorted in the chronological order in which they were established. Senior water rights holders possess indisputable advantage in accessing water resources over junior rights holders [*Hutchins*, 1968, 1977]. Therefore, a given water supply shock affects senior and junior rights holders differently. Prior appropriation allows senior rights holders to secure water supplies first, thereby transferring risk to junior rights holders. Consequently, a macroscale water supply shock such as drought is redistributed unevenly among individual farmers, subsequently affecting their well‐being unevenly. If a water shortage persists, disparity in water allocation among senior versus junior water users may lead to discontent with the water institution and call for change.

The objective of this study is to explore how farmers decide the optimal irrigated acreage when water supply is uncertain, how the prior appropriation doctrine as an example of water institution affects those decisions, and how those decisions subsequently influence farm profit and the shadow value of senior versus junior water rights. We develop an economic model with binding irrigation water constraints. The model builds on *Feder* [1980], who studied a risk‐averse farmer's adoption of new technology under production uncertainty. We modify Feder's framework to explicitly characterize a water institution and uncertainty in irrigation water supply. This setup allows for heterogeneous responses of individual farmers to the same source of water supply uncertainty. More specifically, we model irrigation water supply as stochastic, which generates a portfolio selection problem between risky (i.e., irrigated) and riskless (i.e., nonirrigated) farming activities. Our hypothetical farmers' optimal land allocation is jointly determined by the probability distribution of water supply, the institution that redistributes a limited water supply among individuals based on their water right priority, and the farmers' attitude toward risk. We apply our model to the Eastern Snake River Plain Aquifer, the primary agricultural area in the state of Idaho, where irrigation water allocation follows the prior appropriation doctrine.

This paper makes four contributions to the literature. First, it explicitly models priority‐based water appropriation from a farmer's perspective. While extensive literature has been devoted to efficient water management from the perspective of water institutions [*Fischhendler and Heikkila*, 2010; *Rosegrant and Binswanger*, 1994; *Saleth and Dina*, 2000; *Saleth*, 2004, among others], more work is needed from the standpoint of farmers, akin to *Burness and Quirk* [1979] and *Knox et al*. [2012]. A lack of farm‐level data frequently impedes efforts to address this issue. Using detailed farm‐level data from eastern Idaho, our study analyzes the effects of a water right's priority on farmers' decisions under water supply uncertainty. It also provides an empirical estimate of the shadow value of this priority in eastern Idaho.

Our paper's second contribution lies in a modeling framework that can serve as an alternative to the more common approach of integrating hydrologic, agronomic, and economic equilibrium models at the hydrologic basin scale [e.g., *Rosegrant et al*., 2000]. Our theoretical model represents instead relationships between macroscale hydroclimate risks (which create water supply uncertainty), farmers' risk‐bearing ability under institutional water management, and optimal irrigated acreage. We can derive an analytic solution to our refined model, as a closed‐form expression, in contrast to many studies that use intensive mathematical programming. We also demonstrate heterogeneous responses to water supply uncertainty triggered by senior versus junior priorities, which contributes to a growing effort to depict heterogeneity in water resources management models (e.g., agent‐based modeling) [*Cai*, 2008].

Our paper's third contribution is that it underscores the importance of accounting for potential asymmetry, such as negative skewness or right truncation, of a risk distribution when modeling farmers' land use decisions. The literature on agricultural production risk, particularly for climate or weather, commonly uses first and second central moments (i.e., mean and variance) to characterize uncertainty [e.g., *Deschênes and Greenstone*, 2007; *Lobell et al*., 2014; *Mendelsohn et al*., 1994]. The third moment, which measures asymmetry of a probability distribution and can convey important information about the possibility of severe water shortages when left‐skewed, is generally overlooked, albeit with a few exceptions [e.g., *Antle*, 1987; *Kim et al*., 2014; *Weitzman*, 2009]. Right truncation, which measures downside risk and can characterize the binding water allotments in the western U.S., also deserves a thorough investigation. Our paper demonstrates that both negative skewness and right truncation are, in fact, the key parameters influencing the impact of a water right's priority on land allocation to irrigated and nonirrigated crops. They are also essential in determining the shadow value of a water right's priority.

A fourth contribution is that the model can be applied to a variety of situations that involve risk‐averse behavior under water supply uncertainty when water institutions are present. We demonstrate how to explore the impacts of future water shortages under several climate change scenarios. The ability to adapt our model to various real‐world situations is particularly valuable when microlevel water use data are unavailable for a large geographic scale, necessitating an analytical modeling approach.

The remainder of our paper is organized as follows. Section 2 models the farmer's land use decision in the presence of water scarcity and uncertainty. Section 3 applies the model to a real‐world scenario, using data from eastern Idaho, to illustrate the major propositions derived in the previous section. Section 4 concludes the paper.

## 2 The Model

This section establishes the framework of a formal model of production under water supply uncertainty. We use this model to explore the effect of a water right's allocative priority—as an indicator of a farmer's risk‐bearing ability—on a risk‐averse farmer's decision about how much land to irrigate. The discussion begins with a general model with flexible water allotments. Then we consider a refined case in which binding water allotments are subject to curtailment.

### 2.1 The Case of Flexible Water Allotments: A General Model

Assume a hypothetical farmer owns a farm with fixed permitted irrigated acreage ( $\bar{L}$) and takes prices as given. She allocates land between risky, water‐intensive crops and riskless, drought‐tolerant crops. Simplifying the assumption about the risk of growing drought‐tolerant crops, we concentrate on the impact of water supply risk on water‐intensive crops. Drought‐tolerant crops are susceptible to potential damages caused by local hydroclimate conditions. But water‐intensive crops are more sensitive to the same stress, which could result in larger damages. Also for simplicity, we normalize the price margin (i.e., price minus unit cost) of water‐intensive crops and assume that drought‐tolerant crops have fixed net returns per acre (*R*) regardless of water supply. The farm's production technology exhibits constant returns to scale (CRS) such that total output from water‐intensive crops equals *Ly*(*w*), where *L* is the amount of actual irrigated land, *y* is a yield function, and *w* is water application per irrigated acre. By definition, *y*′> 0 and *y*″< 0, and 0 ≤ *y*(0) < *R*.

Compared with land resources, water resources are scarce enough that the water constraint is binding. We assume that water appropriation follows the priority principle alone, and that temporary market‐based transactions such as water banking have an insignificant impact on regional water allocation. This is because, in practice, water transactions are sparse [*Hadjigeorgalis*, 2009] and often associated with high transaction costs [*Easter et al*., 1999; *Carey et al*., 2002]. We also assume that a farm's water constraint depends on three factors: the expected total water allotment ( $\bar{W}$), uncertain water supplies characterized by a random variable *ɛ* with mean zero and variance *σ* ^2^, and the allocative priority of their irrigation water rights (*V*) which connects the macroscale risk to individual farmers. The farmer's water constraint is written as:

(1) $$wL=\bar{W}+ɛH\left(V\right)\geq 0.$$

Equation (1) implies *w* = [ $\bar{W}$+*ɛH*(*V*)]/*L*, which will enter the utility function later. For the stochastic term *ɛ*, we only consider uncertainty in total water supply at the basin or larger scale. The term *H*(*V*) reflects an individual farmer's risk‐bearing ability, namely their ability to control the impact of water supply risk or transfer the risk to other individuals through a water sharing mechanism. *H* is the same for all farmers and only *V* varies. *H*(*V*) is always positive and decreases with *V*, i.e., *H*′≡*∂H*/*∂V* < 0. A small *H* (a large *V*) is associated with a high risk‐bearing ability. This setup allows us to explicitly connect the institutional water sharing arrangement (i.e., senior versus junior priorities) with a farmer's decisions about how much land is devoted to water‐intensive crops. The macroscale risk *ɛ* generates different impacts on individual farmers, varying with the allocative priority of their irrigation water rights, given the same water source. Senior water rights holders (a large *V*) are less affected by water supply uncertainty than junior holders and thus have a smaller *H*(*V*).

Assume the farmer's objective is to maximize the expected utility (*U*) of income (*π*). We model the utility function as strictly concave (i.e., *U* = *U*(*π*), *U*′> 0, *U*″< 0), reflecting risk aversion. Income is defined as the sum of net revenue from water‐intensive and drought‐tolerant crop production

(2) $$\pi \equiv Ly\left(w\right)+R\left(\bar{L}-L\right).$$

The objective function is then given by

(3) $$\max_{L}EU\left[Ly\left(\frac{\bar{W}+ɛH\left(V\right)}{L}\right)+R\left(\bar{L}-L\right)\right].$$

#### 2.1.1 Optimal Allocation of Land

Maximization of the objective function above with respect to (w.r.t.) *L* requires the following first order condition (FOC):

(4) $$\frac{\partial EU}{\partial L}=E\left[U^{\prime }\left(y-R-y^{\prime }\;\frac{\bar{W}+ɛH\left(V\right)}{L}\right)\right]=0.$$

Denote $y-R-y^{\prime }\frac{\bar{W}+ɛH\left(V\right)}{L}\equiv \Phi$ (note that Φ = ∂*π*/∂*L*). Differentiating Φ w.r.t. *L* yields

(5) $$\Phi_{L}\equiv \frac{\partial \Phi }{\partial L}=y^{\prime \prime }\frac{{\left[\bar{W}+ɛH\left(V\right)\right]}^{2}}{L^{3}}<0.$$

Since Φ is monotonically decreasing in *L*, an interior optimal solution for *L* is unique. If cov(Φ,*U*′) = 0, the optimal land allocation strategy is simply given by setting Φ = 0. We posit, however, that cov(Φ,*U*′) ≠ 0 holds under general situations, which directs the following discussion. From equation (4), one can derive the relationship between the optimal acreage of land irrigated (i.e., water‐intensive crops) and other relevant parameters.

Of particular interest is the effect of allocative priority of an irrigation water right on the optimal land allocation (dL^*^/*dV*), which can be derived by differentiating equation (4) w.r.t. *L* and *V*:

(6) $$\frac{dL^{*}}{dV}=-\frac{E\left(U^{\prime \prime }y^{\prime }H^{\prime }\Phi ɛ+U^{\prime }\Phi_{V}\right)}{E\left(U^{\prime \prime }\Phi^{2}\right)+E\left(U^{\prime }\Phi_{L}\right)},$$

where $\Phi_{V}\equiv \frac{\partial \Phi }{\partial V}=-y^{\prime \prime }H^{\prime }\frac{\bar{W}+ɛH}{L^{2}}ɛ$.

To determine the sign of dL^*^/*dV*, we apply the Taylor series approximation to separate the random terms from the deterministic terms (see Appendix A). The approximation implies that dL^*^/*dV* will be positive if and only if

(7) $$\gamma <\frac{y_{0}-R-3y_{0}^{\prime }\bar{w}}{y_{0}^{\prime \prime }\bar{w}h\sigma },$$

where *γ*≡*E*(*ɛ* ^3^)/*σ* ^3^, measuring the skewness of the probability distribution of the macroscale water supply risk *ɛ*; *y*_0_, *y*_0_′, and *y*_0_″ are deterministic terms evaluated at $\bar{w}$; $\bar{w}$≡ $\bar{W}$/*L*; and *h*≡*H(V)*/*L*. Although the signs of both sides of condition (7) are undetermined, the inequality in (7) will always hold when *γ* is sufficiently small.

This result yields the following proposition:

**Proposition 1**. The optimal acreage of land irrigated (L^*^) increases with water right priority (V) if the probability distribution of ɛ has a strong negative skew (left‐skewed).

The proofs for Proposition 1 and all subsequent propositions and corollaries are provided in Appendix A. Two points arise from this proposition. First, the effect of *V* on L^*^ remains indefinite in general. A positive effect is expected iff the skewness condition is met. Second, when the shock of a severe water shortage is anticipated, a risk‐averse farmer with a small *V* (i.e., a low risk‐bearing ability) will reduce L^*^.

The level of net returns from drought‐tolerant crops *R* also affects L^*^. Differentiating equation (4) w.r.t. *L* and *R* yields:

(8) $$\frac{dL^{*}}{dR}=-\frac{E\left[U^{\prime \prime }\Phi \left(\bar{L}-L\right)-U^{\prime }\right]}{E\left(U^{\prime \prime }\Phi^{2}\right)+E\left(U^{\prime }\Phi_{L}\right)}.$$

It is easy to show that dL^*^/*dR* < 0. This result gives Proposition 2:

**Proposition 2**. The optimal acreage of land irrigated (L^*^) decreases with net returns per acre from drought‐tolerant crops (R).

The intuition from Proposition 2 is straightforward. An increase in the profitability of drought‐tolerant crops will always raise the comparative advantage of these crops relative to water‐intensive crops, assuming the net returns from the two types of crops are not correlated. A net revenue increase for drought‐tolerant crops will subsequently reduce the optimal proportion of land receiving irrigation.

#### 2.1.2 Shadow Value of a Water Right Priority

As demonstrated in the preceding subsection, senior water rights holders adapt more easily to water supply uncertainties than junior rights holders, and tend to irrigate more of their land, assuming water‐intensive crops are more profitable than drought‐tolerant crops. Therefore, a higher priority is preferable to a lower one. This subsection assesses the shadow value of a water right's priority using the indirect utility function, which we derive from the previous subsection. Here the shadow value (*λ*) is defined as a farmer's willingness to pay for an extra improved priority.

The farmer's indirect utility function is given by:

(9) $$U^{*}\left(V,\bar{W},R\right)=EU\left[L^{*}y\left(\frac{\bar{W}+ɛH\left(V\right)}{L^{*}}\right)+R\left(\bar{L}-L^{*}\right)\right].$$

The envelope theorem implies

(10) $$\lambda =\frac{\partial U^{*}}{\partial V}=H^{\prime }E\left(U^{\prime }y^{\prime }ɛ\right).$$

An analogue Taylor series approximation to equation (10) reveals that

(11) $$\lambda ≅H^{\prime }\sigma^{2}\left(U_{0}^{\prime }y_{0}^{\prime \prime }h+U_{0}^{\prime \prime }y_{0}^{\prime }HA\right),$$

where *A*≡*y*_0_′*+y*_0_″*hσγ*. Since *H*′< 0, *H* > 0, *U*_0_′*y*_0_″< 0, and *U*_0_″*y*_0_′< 0, *λ* is positive if (i) *A* is positive, or (ii) *A* is negative and the magnitude of *U*_0_′*y*_0_″*HA* is sufficiently small. Recall that *γ* measures the distributional skewness for the macrorisk *ɛ*. When *γ* < 0 is satisfied, *ɛ* has a left‐skewed distribution, constituting a sufficient condition for a positive *A* and hence a positive *λ*.

Furthermore, when *γ* < 0, it is easy to show that *∂λ*/*∂*|*γ*| > 0. This finding can be summarized as:

**Corollary 1**. *If the probability distribution of the macrorisk ɛ has a negative skew (γ < 0), an increase in the degree of that negative skewness (|γ|) will increase the shadow value of an allocative water right's priority (λ), at equilibri*um.

Intuitively, the shadow value reflects the benefit to the farmer of having a higher priority water right: a higher priority reduces the share of any given macroscale water shortage that the farmer has to bear, and this, in turn, reduces the negative impacts of water shortage on the farmer's crop yields. Of course, the overall magnitude of a water shortage's impact depends on the severity of the macrorisk. The more left‐skewed the macrorisk's distribution is, the more likely a water shortage will be severe, and the larger the potential negative crop yield impacts will be. Finally, the larger the potential negative impacts, the higher premium a farmer is willing to pay to obtain a higher water right priority to hedge against risk.

### 2.2 The Case of Binding Water Allotments: A Refined Model

So far we have presented an optimal land allocation model with a stochastic water allotment, which is jointly determined by the expected water allotment ( $\bar{W}$) and a zero‐mean random variable *ɛ*. In some regions of the western U.S., the water allotment that a water right holder is entitled to withdraw during a prescribed period is binding. Each water right holder has a predetermined, fixed water allotment. In dry years, junior rights holders are likely to receive less water than their allotments due to frequent curtailments; in wet years, they also cannot receive more than their allotments due to the rule of beneficial use [*Hutchins*, 1968, 1977].

To accommodate this situation, we modify the probability distribution of the macrorisk in irrigation water supply such that the risk is *right truncated* at $\bar{w}$. Let *S* be the level of stochastic natural flow of a surface stream, based on which a watermaster determines which groups of water rights holders are eligible to divert water for irrigation. For any given water right holder with priority *V*, her water use on a per‐irrigated‐acre basis is a random variable:

(12) w=w¯, S>sV0, S≤sV,

where *s*(*V*) represents the nonstochastic curtailment level of the flow, below which water rights holders with priority *V* and more junior users are not allowed to withdraw water for irrigation. The curtailment function *s*(*V*) decreases with *V*, i.e., *s*′≡*∂s*/*∂V* < 0, implying that the more senior the priority, the lower the curtailment level of streamflow. Compared with the model setup developed in the previous subsections, the stochastic streamflow *S* replaces the random variable ɛ, used to measure water supply uncertainty at the macroscale; the curtailment function *s*(*V*) substitutes for the function *H*(*V*), used to measure an individual water right holder's risk‐bearing ability.

Given the modified probability distribution of *w* in equation (12), we explicitly expand the farmer's expected utility function and rewrite her objection function as

(13) $$\max_{L}\left(1-F_{S}\left[s\left(V\right)\right]\right)U\left[Ly\left(\bar{w}\right)+R\left(\bar{L}-L\right)\right]+F_{S}\left[s\left(V\right)\right]U\left[Ly\left(0\right)+R\left(\bar{L}-L\right)\right],$$

where $F_{S}\left[s\left(V\right)\right]\equiv Pr\left[S\leq s\left(V\right)\right]$, representing the cumulative distribution function of *S*. Analogous to the previous analysis, Φ(*w*) is defined as the derivative of income *π* w.r.t. irrigated land *L*, i.e., Φ(*w*)≡*y*(*w*) − *R* − *y*′(*w*)*w*.

Maximization of objective function (13) w.r.t. *L* requires:

(14) $$\frac{\partial EU}{\partial L}=\left(1-F_{S}\left[s\left(V\right)\right]\right){\left(U^{\prime }\Phi \right)}_{|w=\bar{w}}+F_{S}\left[s\left(V\right)\right]{\left(U^{\prime }\Phi \right)}_{|w=0}=0.$$

We can still demonstrate that Φ is monotonically decreasing in *L* ( $\Phi_{L}=y^{\prime \prime }\frac{{\bar{W}}^{2}}{L^{3}}<0$), implying a unique interior optimal solution for *L*. Differentiating equation (14) w.r.t. *L* and *V* gives

(15) $$\frac{dL^{*}}{dV}=\frac{s^{\prime }}{1-F_{S}\left[s\left(V\right)\right]}{\left.\left(\frac{U^{\prime }\Phi }{U^{\prime \prime }\Phi^{2}+U^{\prime }\Phi_{L}}\right)\right|}_{w=\bar{w}},$$

implying that *dL* ^*^/*dV* > 0, $\frac{d}{d\left|s^{\prime }\right|}\left(\frac{dL^{*}}{dV}\right)$>0, and $\frac{d}{dF_{S}\left[s\left(V\right)\right]}\left(\frac{dL^{*}}{dV}\right)$ >0 (see proofs in Appendix A).

Likewise, the shadow value of a water right's priority can be derived by applying the envelope theorem to the farmer's indirect utility function

(16) $$\lambda =-s^{\prime }f_{S}\left[s\left(V\right)\right]\left(U_{|w=\bar{w}}^{*}-U_{|w=0}^{*}\right),$$

where $f_{S}\left[s\left(V\right)\right]$ is the probability density function of *S* and $F_{S}\left[s\left(V\right)\right]=\int_{0}^{s\left(V\right)}f_{S}\left(u\right)du$. Since *s* ′<0, *f_s_*[*s*(*V*)] > 0, and $U_{|w=0}^{*}$< $U_{|w=\bar{w}}^{*}$ (because $\frac{\partial U}{\partial w}=U^{\prime }y^{\prime }L$>0 is evident), it is easy to show that *λ* > 0, *∂λ*/*∂*|*s* ′| > 0, and *∂λ*/*∂* $f_{S}\left[s\left(V\right)\right]$ > 0.

In theory, the shadow value is determined by two factors. The first factor is the effectiveness of the priority in coping with the risk of being curtailed, i.e., |*∂F_s_*[*s*(*V*)]/*∂V*|, which can be further simplified to |*s* ′ *′f_s_*[*s*(*V*)]|. The more effective a higher water right priority, the larger the shadow value. The second factor is the difference in utility between receiving a full water allotment and no allotment, both evaluated at L^*^. The utility difference generally increases with *V* because a more senior water right leads to a higher L^*^, and this, in turn, implies a larger $U_{|w=\bar{w}}^{*}$ and a smaller $U_{|w=0}^{*}$ (recall that $U_{|w=x}^{*}\equiv U\left[L^{*}y\left(x\right)+R\left(\bar{L}-L^{*}\right)\right]$ and *y*(0) < *R* < *y*( $\bar{w}$)). Yet without knowing exactly how a water right's priority affects the marginal change in propensity of curtailment on a particular stream, the quantitative effect of *V* on *λ* is generally indeterminate.

The above discussion can be summarized as:

**Proposition 3**. With a nonstochastic, binding water allotment, the optimal acreage of land irrigated (L^*^) always increases with the priority of a water right (V). The effect of V on L^*^ increases with the probability of curtailment (F_s_[s(V)]) and with the sensitivity of the curtailment level of streamflow to a water right's priority (|s′|).

**Corollary 2**. With a nonstochastic, binding water allotment, the shadow value of a water right's priority (λ), at equilibrium, is always positive. The value of λ increases with the sensitivity of the curtailment level of streamflow to a water right's priority (|s′|), the probability of streamflow falling into a range arbitrarily close to the curtailment level (f_s_[s(V)]), and the difference in the utility between full use and nonuse of the water allotment, evaluated at L^*^.

Analogous to the model developed in the previous subsections, the model presented here still captures the role of allocative priority water rights in redistributing water supply risk among farmers. Importantly, Proposition 3 supports Proposition 1 by presenting a special case, in which the macrorisk ɛ is right truncated at zero, to better approximate the real‐world water institution. In the context of this study, a distribution of ɛ that is right truncated at zero is always left‐skewed. Thus, Proposition 3 retains the full intuitions of Proposition 1.

## 3 An Application

This section presents a case study, using a simulation approach to illustrate the application of the binding‐water‐allotment model in the Eastern Snake River Plain Aquifer (ESPA) region of eastern Idaho. Located along the Snake River in arid and semiarid climate zones, ESPA is the prime agricultural region in Idaho and relies heavily on irrigation water supplies during the growing season. Irrigated agriculture accounts for approximately 80% of total water withdrawals in the 21 counties that encompass the ESPA region [*Maupin et al*., 2014]. Farmers in the ESPA region grow a wide array of crops, including alfalfa, barley, dry beans, corn, hay, onions, potatoes, sugar beets, and wheat (durum/spring/winter).

The simulation explores the relationship between water right priority, water supply uncertainty, and land irrigation decision under alternative climate change scenarios. It elaborates the complex relationship between water right priority and its shadow value when curtailment occurs. The heterogeneity in farm performance between senior versus junior water right holders is also addressed. The analysis could facilitate an improved understanding of the profitability of farm and the sustainability of irrigated agriculture under climate change.

Specifically, this case‐study application enables us to explore the following questions:

1. What are the effects of water right priority on a representative farmer's optimal land irrigated, given water supply uncertainty and assuming zero development costs? How does the shadow value of water rights priority change with the priority?
2. For a representative farmer, how would water supply uncertainty into the future affect her optimal irrigated acreage, annualized expected net revenue, and lump sum shadow‐value of her water right?
3. How do junior and senior rights holders differ in their land irrigation decisions and associated economic returns? How would these disparities change under various future hydroclimatic scenarios?

### 3.1 Water Rights Management in Idaho

When applying the theoretical model for empirical analysis, it is important to understand how a particular water resource is managed in practice. Like many parts of the U.S. West, the ESPA region follows the prior appropriation doctrine to allocate water to beneficial uses under the Idaho law of water rights [*Bretsen and Hill*, 2009; *Hutchins*, 1968, 1977; *Thompson*, 1993]. Every water right is described by a set of specific elements, including source, priority date, amount, period of use, purpose of use, point of diversion, and place of use.

Priority date is the date on which a person (or her predecessor) first began using the water for a beneficial use. On the basis of “first in time, first in right,” the earlier (i.e., more senior) the priority date, the more secure the water right, regardless of where the user is located on a stream. When the streamflow declines to a predetermined level, a state agent known as a watermaster closes the headgates to the irrigation canals of the most junior rights holders. When the flow drops further, the watermaster closes the headgates of the next most junior rights holders, and so forth. The most senior water right holder is the last to be affected by curtailment, and typically only when a water shortage is especially severe.

All water rights are quantified in either annual volume or flow rate, or both. Very early irrigation water rights are quantified simply by reference to the acres of land that are irrigated. Older water rights are often quantified by a diversion rate (usually not more than 0.02 cubic feet per second (cfs) per irrigated acre). The annual volumetric limit (acre feet or a.f.), if not specified, is estimated based on historical use. Today, new water rights typically specify a diversion rate, period of use, and annual volume (i.e., annual cap) [*Hutchins*, 1968; *Fereday et al*., 2010, p. 6].

In Idaho, if a farmer diverts less water than their annual cap, their water right will not be reduced unless the right is transferred to a new use (e.g., from agricultural use to municipal use). In other words, as long as the water right continues to be used for irrigation, a farmer retains the flexibility to convert back and forth among irrigation systems or among more or less water‐intensive crops without the risk of having their rights cut back [*Hutchins*, 1968; *Fereday et al*., 2010, p. 7]. If, however, a farmer seeks to transfer her water right to a new type of use, the Idaho Department of Water Resources (IDWR) evaluates the quantity of water available for transfer based on the farmer's recent historical use typically over the last 5 years.

A closely related issue is the conditions that can lead to a farmer losing their water rights permanently. In theory, failure to use all or part of a water right for the last 5 years can result in forfeiture of the right. But this rule does not apply when the nonuse results from circumstances beyond the right holder's control, or if there is no need to divert water due to wet weather conditions [*Hutchins*, 1968; *Fereday et al*., 2010, p. 21]. Farmers can also avoid forfeiting a portion of their water right by placing it in a “water bank” or “rental pool” that is established pursuant to state law [*Fereday et al*., 2010, p. 24].

### 3.2 Model Parameterization

The refined model with binding water allotments requires a set of representative parameter values for total permitted irrigated acreage, actual irrigated acreage, water rights priority dates, water allotments, production functions for water‐intensive crops, price margins for water‐intensive crops, net revenues for drought‐tolerant crops, and risk measurements for the ESPA region. Most data used to parameterize the model were extracted from a comprehensive geodatabase [*Xu et al*., 2014]. The database contains detailed information on irrigation status, priority dates, crop varieties, crop water use intensity, crop yields and prices, and other agriculturally relevant variables from various sources, including the Water Rights Geospatial Layers from the IDWR [*Idaho Department of Water Resources (IDWR)*, 2010] and annual Cropland Data Layers from the U.S. Department of Agriculture, National Agricultural Statistics Service for 2009 through 2011 [*U.S. Department of Agriculture, National Agricultural Statistics Service (USDA‐NASS)*, [Link]a]. These variables were standardized to the farm level as reported in *Xu et al*. [2014]. We focus on farms that rely primarily on surface water for irrigation and actually practiced irrigation during this period. The resulting panel data set consists of 2227 farms with an average land area permitted for irrigation ( $\bar{L}$) of 395 acres, and an average actual irrigated area (*L*) of 287 acres, implying that 72.6% of permitted land is actually irrigated under baseline conditions.

For each farm, we identified the dominant establishment date of its water rights and normalized that date uniformly on a scale of 0 to 1, for technical simplicity. The value 0 represents the most junior right, with the date of September 2009; the value 1 represents the most senior right with the date of January 1870. The average normalized establishment date in the sample is 0.674 (equivalent to the date of July 1915). We use this simplified measure to proxy *V*, the water right priority.

The average annual fixed water allotment (i.e., annual cap) $\bar{w}$ for an irrigator in EPSA is 5.316 a.f. acre^−1^. We base this value on Idaho's typical allotment for surface water rights of 0.02 cfs per irrigated acre, equivalent to 0.0397 a.f. acre^−1^ d^−1^. The daily water allotment is then multiplied by 134 days, the average growing season length for major crops in the region [*USDA‐NASS*, 2010].

As a robustness check, we also estimate the potential maximum water allotment for each farm in our sample. On average, the potential maximum water allotment was 6.681 a.f. acre^−1^ during 2009–2011. To arrive at this estimate, we divided actual water use intensity by the noncurtailment rate during the growing season. Actual water use intensity was calculated by taking a weighted average of water use intensity for each major crop (weighted by that crop's irrigated area) as reported in the Crop Water Use Information of AgriMet [*U.S. Department of Interior*, [Link]]. We calculated the noncurtailment rate using the IDWR's weekly records on curtailment priority dates [*IDWR*, [Link]]. We conclude that our use of the average annual fixed water allotment parameter of 5.316 a.f. acre^−1^, rather than the maximum allotment of 6.681 a.f. acre^−1^, is feasible for a representative farm considering any remaining uncertainties in farm‐level details such as water losses during conveyance and cropping patterns.

To generate annual gross revenue and production costs for irrigated and nonirrigated crops on each farm, we use state‐level crop prices (which the USDA‐NASS surveys each year) from 2009 to 2011 (USDA‐NASS, [Link]b), and county‐level production expenses data (which the USDA‐NASS reports every five years through the U.S. Census of Agriculture) from 2012. We combine these data with our comprehensive geodatabase's farm‐level, crop‐specific land areas and production output by irrigation status. Because of the short time‐span of the data, we use nominal crop prices in our calculations instead of adjusting for inflation.

Using “crops under irrigation” as a proxy for water‐intensive crops, and using “crops under nonirrigation” as a proxy for drought‐tolerant crops, we calculate two variables: the price margin of water‐intensive crops (i.e., price minus unit cost), and the net revenue from drought‐tolerant crops. Their farm‐level means are ∼$59.40 ton^−1^ and ∼$91.70 acre^−1^, respectively. Detailed calculations of the price margins and net revenue are presented in the supporting information Text S1.

The case‐study simulation requires an explicit formulation for the production functions of water‐intensive crops. We assume a Cobb‐Douglas production function with water and land as inputs—the two most relevant factors in the model—leaving all other inputs to be captured in the coefficient that represents total factor productivity. The Cobb‐Douglas production function is written generally as *Y = aW^ρ^L* ^1−^ ^*ρ*^. Output *Y* is the sum of the production outputs of all major water‐intensive crops, measured consistently in winter wheat‐equivalent output. Land input *L* is the actual irrigated area of a farm. Irrigation water application *W* is estimated by multiplying total irrigated area by the aforementioned actual water use intensity. This estimation is necessary because irrigation application or diversion data in Idaho are generally unavailable at the farm level due to privacy concerns.

Taking the logarithm of the production function on both sides, and using the 3 year panel data set (2009–2011), we estimate the elasticity of yield to water input (*ρ*) as 0.06524 (*p* value < 0.001; *R*‐squared = 0.941; see in the supporting information Figure S1). This value is consistent with estimates in the literature, which generally suggest that yield is relatively in elastic to water input in agricultural production [*Moore et al*., 1993; *Frisvold and Konyar*, 2012; *Frija et al*., 2014]. We do not report the total factor productivity *a* here because it will be integrated in another scalar *b* during the calibration process at the end of this subsection.

Parameterizing the risk component of our model requires measurements at both the macroscale and individual farm level. To parameterize the probability distribution of the macrorisk, we collect daily natural flow (*S*) in cfs and the corresponding lowest priority to receive water from the Water Rights Accounting data for the Heise site for the period 2009–2011 [*IDWR*, [Link]]. The Heise site is chosen because the streamflow at this site serves as a good indicator of water supplies for ESPA and the State of Idaho. We record these data every week from 1 April through 30 September, during which irrigation withdrawals are generally permitted by law. These rich data enable us to generate a smooth curve for *s*(*V*)—the relationship between priority date and the corresponding curtailment level of streamflow.

Assuming *s*(*V*) is an exponential function of *V*, we regress ln(*S*) on *V* with various polynomial specifications and choose the one of best fit (*R*‐squared = 0.945 or better):

(17) ln⁡sV=9.72−2.04V+0.89V2, 0.0183<V<0.779.86−10.10V+23.70V2−16.04V3, elsewhere.

The inverse relationship between *V* and *s*(*V*) is confirmed by the empirical data. The piecewise functional form in (17) allows us to capture the structural features of the most junior and most senior water rights holders (see supporting information Text S2 and Figure S2). Given the recorded daily streamflow *S* and the curtailment function *s*(*V*), we can calculate the propensity of curtailment (*F_s_*[*s*(*V*)]) for any water right holder during the period 2009–2011. For example, for a farm with an average priority (*V* = 0.674), *F_s_*[*s*(0.674)] = 0.381. This estimated value is quantitatively close to both the corresponding recorded curtailment frequency (0.417) and the recorded frequency averaged across all farms in our data sample (0.378).

In addition to the historical natural flows recorded by the IDWR, we also employ the daily streamflow projections for the future years 2017 through 2098, as generated by the Variable Infiltration Capacity (VIC) hydrologic model using data from two general circulation models (GCMs): CnrmCM3 and HadCM, each under the two different emission scenarios, A1B and B1. The VIC model is one prominent product from the Columbia Basin Climate Change Scenarios Project, which has been widely used in the hydrologic literature [*Hamlet et al*. 2010]. The VIC model generates hydrologic output variables, including streamflow, under 14 different GCM scenarios. Among them, the A1B emissions scenario forecasted by the CnrmCM3 is closest to the daily recorded historical streamflow at Heise for the period 2009–2011. The CnrmCM3 is the third version of the ocean‐atmosphere model initially developed by the European Center for Advanced Research and Training in Scientific Computing and then regularly updated by the Center National Weather Research (CNRM). The correlation coefficient of daily streamflow between the hydrologic simulation and the historical record is ∼0.635. We calibrate the VIC streamflow to remove the systemic bias of hydrologic simulation against the historical streamflow. Details about the data and the calibration procedure are presented in the supporting information Text S3 and S4.

One last step in the parameterization of our model for risk is to assume a form and magnitude of risk aversion for our representative farmer. We assume she exhibits constant relative risk aversion, and thus specify her utility function as *U*(*π*) = *π* ^1−^ ^*α*^/(1−*α*). Following *Feder* [1980], we set the coefficient of risk aversion (*α*) at 0.5. Finally, we solve for the scalar term (*b*) of the production function by submitting all baseline values of the parameters reported in Table 1 to the FOC from equation (14). The term *b* captures all omitted covariates in the simplified production function. Our calculation indicates that *b* takes the value of 4.229.

**Table 1:** Characteristics of Land and Water Use, Production, and Utility

Parameters	Symbols	Values	Sources
*Land and Water Use*			
Average actual irrigated land	*L*	287	Measured in acres, calculated from the database compiled by *Xu et al*. [2014]
Average permitted irrigated land	$\bar{L}$	395	Measured in acres, calculated from the database compiled by *Xu et al*. [2014]
Price margin of water‐intensive crops	*p*	59.4	Measured in $ ton^−1^, calculated from the database compiled by *Xu et al*. [2014]
Net revenue from drought‐tolerant crops	*R*	91.7	Measured in $ acre^−1^, calculated from the database compiled by *Xu et al*. [2014]
Water allotment per permitted acre	$\bar{w}$	5.316	Measured in a.f. acre^−1^, calculated based on the average length of growing period of major crops [*USDA‐NASS*, 2010]
*Allocative Priority of Water Rights*			
Average allocative priority index	*V*	0.674	Calculated from the database (0–1)
Curtailment function	*s*(*V*)	See equation (17)	Estimated from the Water Rights Accounting information [*IDWR*, [Link]]
*Production Function*			
Preference (output elasticity) of water input	*ρ*	0.06524	Estimated from the production function of water‐intensive crops
Scalar	*b*	4.229	Solved from the FOC
*Utility Function*			
Arrow‐Pratt measure of relative risk aversion	*α*	0.50	Set as in *Feder* [1980]

### 3.3 Simulation

This subsection analyzes the effects of water rights priority on optimal proportion of land irrigated under various hydroclimate scenarios. Specifically, we use the recorded streamflow from 2009 to 2011 as the baseline scenario, representing the status quo. We treat the 2009–2011 as a single period and calculate the curtailment probability based on the frequency of a water right being curtailed during these three years. This helps us fully use the information on the relationship between streamflow and curtailment embedded in the existing data. Four alternative scenarios are considered for the future from 2017 to 2098, where each year is treated individually and daily streamflow is predicted by the VIC model based on hydroclimate projections forecasted by the two GCMs, CnrmCM3 and HadCM, under the A1B and B1 emissions scenarios (hereafter CnrmCM3‐A1B, CnrmCM3‐B1, and so forth). We calculate the curtailment probability separately for each year and discount the future value of pecuniary variables using a discount rate of 5% per annum.

#### 3.3.1 Effects of Water Right Priority

**Figure 1:**
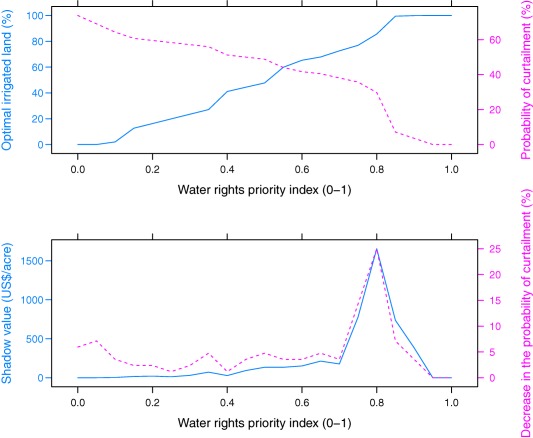
The effects of water rights priority under the baseline scenario. Source: Authors.

We first solve the representative farmer's optimization problem in (13) by changing the water rights priority index *V* from 0 to 1 under the baseline scenario. Then we estimate the expected net revenue using equation (2) and calculate the shadow value of water rights using equation (16), assuming a marginal improvement of 10 years in priority date. Shadow value is measured in present lump‐sum form over an infinite period. Because water rights are real properties, their values reflect the total present value of marginal benefits from a 10‐year improvement in every individual period. Simulation results are illustrated in Figure 1. The top plot corresponds to the effects of water right priority on the probability of curtailment and the optimal proportion of land irrigated. The bottom plot depicts the effects of priority on the marginal decline in curtailment probability in response to a 10‐year improvement in priority date, and the corresponding shadow value.

Consistent with Proposition 3, the optimal proportion of land irrigated increases with the water right priority. Under the baseline, the most junior rights holders (*V* ≤ 0.05) face a high chance of curtailment (69.0–73.8%). Consequently, junior water users no longer grow water‐intensive crops. In sharp contrast, the most senior water users (*V* ≥ 0.95) have no risk of being curtailed so they allocate all their permitted land to water‐intensive crops. The difference in irrigation decisions between the most senior and the most junior users translates to a gap in annual expected net revenue of $188.4 acre^−1^ or $74,409 per farm. This gap is 2 times as much as the annual expected net revenue of the most junior farmers, and almost equivalent to the annual expected net revenue of farmers from the middle priority group (*V* is around 0.50).

The simulation results also suggest a nonmonotonic relationship between water right priority and its shadow value. As illustrated by the bottom plot of Figure 1, moving along the horizontal axis, the shadow value slowly increases with *V* before *V* approaches 0.70; beyond this point, the slope of the shadow value deepens and the shadow value reaches the highest value of $1,654 acre^−1^ at *V* = 0.80 (i.e., priority date of December 1897).

The general pattern of curtailment in water rights management in the ESPA region aligns with this finding. A watermaster usually opens the headgates of farmers from the middle and senior priority groups (*V* > 0.50–0.63) in April and May. Less‐senior rights holders (*V* < 0.50) may only have access to irrigation water in June and July; the duration depends on streamflow in that particular year, typically ranging from 3 weeks to more than 2 months. Around mid‐July, the watermaster closes the headgates of most farmers except those from the more senior group (*V* > 0.74). In another 1–3 weeks, only farmers with *V* > 0.80 have access to irrigation water.

It is the marginal decline in curtailment probability, resulting from a marginal increase in *V*, that shapes the curve of shadow value versus priority (Figure 1, bottom plot). Here a 10‐year improvement in priority date (Δ*V*≈0.0715) is insufficient to help junior rights holders reduce their curtailment probability. This priority increase also does not contribute to any increase in the irrigated proportion of land or net revenue of the most senior rights holders, because those farmers always have access to irrigation water during the entire growing season. In stark contrast, such an increment is sufficiently large for a farmer with *V* = 0.74 to be upgraded to the most senior group, which can prolong the average irrigation duration by approximately 8 weeks and reduce curtailment probability by 28.6%.

#### 3.3.2 Water Supply Uncertainty in the Future

In addition to examining the effect of water right priority under the baseline with status quo risk of water shortage, we investigate how water supply uncertainty in the future would affect farmers' decision making of land irrigation and associated economic returns. This will inform policymakers about farmers' likely responses to growing climate volatility. Because the streamflow generated under scenario CnrmCM3‐A1B is closest to the recorded historical flow in the study area during 2009–2011, we focus our discussion on this scenario's projections for the future. The other three are used to explore whether the simulation results are sensitive to the other combinations of GCM and emission scenarios. Aligning to the baseline scenario, we report expected net revenue in annualized value and evaluate the present lump‐sum shadow value over an infinite period (instead of the 82 years). Details about the discounting approach are presented in supporting information Text S5.

**Figure 2:**
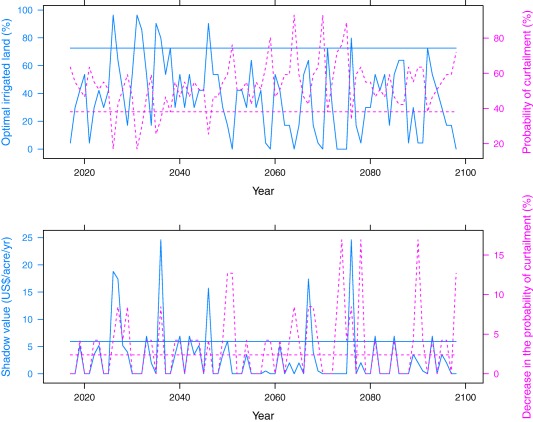
A representative farm's irrigation characteristics under the CnrmCM3‐A1B scenario (*V* = 0.674). Source: Authors. Note: The horizontal lines represent the corresponding baseline level. The shadow value presented here is an *undiscounted* value on an annual basis.

**Figure 3:**
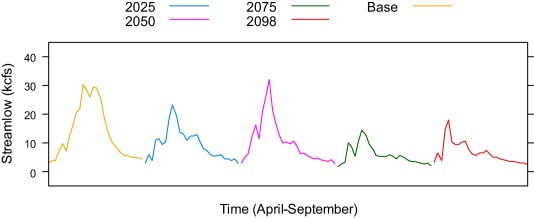
The VIC streamflow prediction for the Heise site under the CnrmCM3‐A1B scenario Source: Authors' calculation based on data from the *IDWR* [2009–2011] and *Hamlet et al*. [2010].

The simulation results, presented in Figure 2 and Table 2, can be summarized in three points. First, under the CnrmCM3‐A1B scenario, farmers might face elevated irrigation water supply risk in the future. Overall, annual streamflow during the growing season declines over time, despite short‐run interannual fluctuations between increases and decreases in streamflow (Figure 3). For a representative farmer at *V* = 0.674, the probability of curtailment will be higher than that under the baseline scenario in 70 out of 82 years (Figure 2). This leads to a nearly 50% contraction in proportion of land irrigated, from the status quo level of 287 acres (72.6%) to an average of 147 acres (37.2%) in the future (Table 2). Such a decline translates to an annualized loss in expected net revenue of approximately $29.50 acre^−1^ or $11,636 per farm. The present lump‐sum shadow value of water right priority would also decline—that is, from $118.20 acre^−1^ in the status quo case to $70.70 acre^−1^ in the future under the CnrmCM3‐A1B scenario. Because shadow value is dictated by the marginal reduction in curtailment probability resulting from an extra improved priority, such marginal reduction under the CnrmCM3‐A1B is zero in almost half of the time, as illustrated in the bottom plot of Figure 2. This implies that for the representative water right, a 10‐year improvement in priority date will not be as effective at reducing the propensity of being curtailed.

**Table 2:** A Representative Farm's Irrigation Characteristics by Hydroclimate Scenarios

			Acre‐Based Value (US$/acre)		Farm‐Based Value (US$1000)	
	Probability of Curtailment (%)	Optimal Irrigated Land (%)	Annualized Expected Net Revenue	Present Lump‐Sum Shadow Value	Annualized Expected Net Revenue	Present Lump‐Sum Shadow Value
Scenario	(1)	(2)	(3)	(4)	(5)	(6)
Baseline (status quo)	38.1	72.6	151.0	118.2	59.6	46.7
CnrmCM3‐A1B	53.1	37.2	121.5	70.7	48.0	27.9
CnrmCM3‐B1	46.6	54.4	145.3	153.0	57.4	60.4
HadCM‐A1B	33.2	75.0	157.6	125.9	62.2	49.7
HadCM‐B1	36.2	71.3	159.8	153.1	63.1	60.5

Note: (i) We set *V* = 0.674 for a representative farm. (ii) By definition, the shadow value is the marginal utility of a farmer for an extra improved priority (10 years). To facilitate the discussion, we convert the marginal utility to US$ by dividing *λ* by ∂*U*/∂*π* when reporting the shadow values in this table. (iii) Columns 3–6 correspond to the present value, evaluated with a 5% discount rate per annum over an infinite period.

Second, the simulation results are *seemingly* sensitive to the choice of a GCM. As presented in Table 2, the average curtailment probabilities and average proportions of land irrigated under the two emission scenarios of the HadCM are quantitatively close to the baseline levels. The annualized expected net revenue and present lump‐sum shadow value are higher than the baseline levels. These disparities in simulation results arise largely from differences in the dynamic patterns of streamflow projected by the two GCMs, more‐so than the choice of emission scenario under the same GCM. For instance, the CnrmCM3 predicts streamflow to decline gradually over time under both emissions scenarios, but such evidence is absent under the HadCM's projected scenarios (supporting information Figure S4).

Third, a larger average proportion of land irrigated is not necessarily associated with a higher annualized expected net revenue. For example, the HadCM‐B1 scenario predicts an average smaller proportion of land irrigated (71.3% versus 75.0%) and a higher annualized expected net revenue ($159.8 acre^−1^ versus $151.0 acre^−1^) than the HadCM‐A1B scenario does. In this case, there are big differences in the temporal distribution of streamflow earlier and later years under the two scenarios. This leads to a lower average curtailment probability (33.7% versus 40.0%), a higher average irrigation proportion (75.9% versus 64.1%), and a higher average annual net revenue ($167.9 acre^−1^ versus $147.9 acre^−1^) in the first 20 years of 2017–2098, followed by a higher curtailment probability (37.0% versus 31.0%), a lower irrigation proportion (69.8% versus 78.6%), and a lower annual net revenue ($156.7 acre^−1^ versus $178.2 acre^−1^) on average in the remaining years, under the HadCM‐B1 scenario than under the HadCM‐A1B scenario. The annual revenues gained over the first 30–40 years determine the annualized revenue (accounting for 77–88%) because the revenues gained beyond this time range will become literally zero‐valued when discounted to the present. Consequently, the annualized expected net revenue is higher under the HadCM‐B1 scenario than under the HadCM‐A1B scenario.

#### 3.3.3 Heterogeneity in Farm Performance with Different Water Right Priorities

We have conceptually established that water right priority may affect the proportion of land a farmer irrigates. We have also evaluated this impact empirically, under current versus future projected climate scenarios. Considering the substantial heterogeneity among different water rights holders, it could be useful to know how water supply uncertainty in the future might affect senior versus junior rights holders differently, specifically how large the disparity in farm performance might be.

To address these questions, we select two groups: one comprising junior rights holders with priority index less than 0.25 (group mean = 0.198), and the other group comprising senior rights holders with priority index greater than 0.75 (group mean = 0.846). We recalculate the same six variables reported in Table 2, while keeping all other parameters unchanged. This simplified assumption allows us to identify the disparity caused solely by heterogeneous rank in water right priority. Table 3 presents the simulation results, where the upper panel corresponds to the junior rights group and the lower panel reports the disparities between senior and junior groups.

**Table 3:** Disparities in the Impacts of Water Supply Uncertainty Between the Junior and Senior Priority Groups

			Acre‐Based Value (US$/acre)		Farm‐Based Value (US$1000)	
	Probability of Curtailment (%)	Optimal Irrigated Land (%)	Annualized Expected Net Revenue	Present Lump Sum Shadow Value	Annualized Expected Net Revenue	Present Lump Sum Shadow Value
Scenario	(1)	(2)	(3)	(4)	(5)	(6)
*Junior Rights Holders (V < 0.25)*						
Baseline (status quo)	59.5	16.2	95.2	21.3	37.6	8.4
CnrmCM3‐A1B	75.7	5.3	94.7	18.8	37.4	7.4
CnrmCM3‐B1	65.9	13.0	97.2	42.4	38.4	16.8
HadCM‐A1B	60.9	20.6	98.6	40.5	38.9	16.0
HadCM‐B1	56.6	27.0	105.2	70.0	41.6	27.7
*Deviation of Senior Rights Holders (V > 0.75) from Junior Rights Holders*						
Baseline (status quo)	−45.2	81.3	141.3	1306.0	55.8	515.9
CnrmCM3‐A1B	−45.7	74.3	96.7	1523.2	38.2	601.6
CnrmCM3‐B1	−35.7	68.2	93.9	1007.9	37.1	398.1
HadCM‐A1B	−47.3	75.4	129.8	1246.5	51.3	492.4
HadCM‐B1	−36.9	65.5	108.4	1367.2	42.8	540.0

Note: (i) To facilitate the discussion, we convert the marginal utility to US$ by dividing *λ* by ∂*U*/∂*π* when reporting the shadow values in this table. (ii) Columns 3–6 correspond to the present value, evaluated with a 5% discount rate per annum over an infinite period.

Focusing first on the upper panel of Table 3—the junior rights group—and on the two emission scenarios under the CnrmCM3, we see that junior rights holders are more likely to be curtailed in the future due to generally lower streamflow than in the baseline. This leads to significant reductions in the long‐term proportion of land irrigated, from 16.2% to 5.7% under the A1B scenario and to 13.0% under the B1 scenario. But the annualized value of expected net revenue under the B1 scenario would be slightly higher than in the baseline, resulting primarily from lower curtailment probabilities and higher economic returns realized in earlier years under the B1 scenario. In contrast, junior rights holders tend to allocate a higher proportion of land for irrigation under the HadCM emission scenarios than in the baseline, leading to an increase in the expected annualized net revenue.

Turning next to the lower panel of Table 3, the simulation results show considerable disparities between the junior and senior groups, in both optimal irrigated acreage and annualized net revenue, and for both the baseline scenario and climate change scenarios. Under the baseline scenario, senior rights holders allocate 81.3% more of their permitted irrigated land to water‐intensive crops than junior rights holders. This enables the senior group to earn about 1.48 times more than the junior group. The gap in annualized expected net revenue can be as wide as $141.40 acre^−1^ or $55,800 per farm. This gap narrows in all future scenarios, ranging from $93.90 acre^−1^ under the CnrmCM3‐B1 scenario to $129.80 acre^−1^ under the HadCM‐A1B scenario. The narrowing trend results primarily from a decrease in the senior group's annualized net revenue in those scenarios compared with the baseline.

Heterogeneity in the lump‐sum shadow value among different priority groups demonstrates a mixed pattern, which adds to the complexity of interpreting projections under different emissions scenarios forecasted by different GCMs. The heterogeneity results primarily from interactions between the change in total volume of streamflow and its intra‐annual distribution during the growing season, leading to different curtailment situations for farmers with different water right priorities. Among other things, the shape of the probability distribution of forecasted streamflow *S* and the critical flow value to initiate curtailment are essential to determine the shadow value of water right priority in the future.

### 3.4 Remaining Modeling Issues

Several issues remain to be resolved relating to the complexity of model formulation, the reliability of theoretical results and projections, and the challenges of its application to real‐world environments.

We have taken multiple steps to simplify the hydrologic components of our model to address model‐solving complexities [*Cai et al*., 2003]. But we nevertheless attempt to strike a balance between the complexity of hydrological processes and the feasible application of our model to real‐world environments, which is important for holistic modeling [*Cai*, 2008]. We embed relatively simple hydrological processes and more sophisticated economic components into one consistent modeling framework, and simplify the curtailment function by assuming it static.

Another simplifying hydrologic assumption is the absence of groundwater in our model, despite the presence of groundwater in our study area. In a separate empirical study, we find that groundwater can provide an alternative mechanism to mitigate the impact of water shortage risk, which substantially complicates the overall measure of risk‐bearing ability. Consequently, the incorporation of groundwater allocation in future versions of our model would contribute to better policymaking, particularly regarding conjunctive management in the face of water shortages.

We focus the bulk of our modeling efforts on characterizing a farmer's land use decisions in the face of water shortage risks under a water institution. Consequently, our model includes relatively complex constraints on water use and land allocation to better reflect the prior appropriation doctrine, something few economics models have adequately captured. But within the economic components of our model, we still have made some simplifying assumptions. For example, we simplify the production process by only accounting for total water application during the growing season, abstracting away from intraseasonal timing of those applications and their effects on crop yields. This would require extensive agronomic data and modeling that go beyond the scope of our study. We also simplify our model of farmer behavior by assuming they are rational decision makers who have perfect information about the properties of the water allotment probability distribution. We do not explore how our findings could be affected by sequential learning dynamics, which is extensively addressed in *Millner* [2009].

## 4 Conclusions

This paper explores the impacts of a water right's allocative priority—as an indicator of farmers' risk‐bearing ability—on the acreage of land irrigated under a status quo macroscale risk of water shortage as well as under future climatic conditions. A theoretical model is developed and applied to the ESPA region of Idaho. The paper reveals several useful insights.

First, a water right's priority significantly affects a farmer's land irrigation decision. The optimal irrigated acreage increases with water right priority when the macroscale water supply risk features a left‐skewed or right‐truncated distribution, indicating the possibility of severe water shortage or downside risk. Using highly detailed empirical data from ESPA, we find considerable disparities in the proportion of land irrigated between junior and senior water rights holders. Senior rights holders are able to irrigate almost all their permitted land, 5 times more than junior rights holders can. This disparity leads to a wide gap—reaching up to $141.4 acre^−1^ or $55,800 per farm—in annual expected net revenue, equivalent to the revenue of farmers having middle‐level priority rights.

Second, our simulation results of irrigated land‐use decisions under future climatic conditions are relatively robust across emissions scenarios under the same GCM, but are *seemingly* sensitive to the choice of a GCM. The CnrmCM3‐A1B scenario suggests that the proportion of land irrigated on a representative farm in the EPSA would decrease by half from the status quo level of 72.6% (287 acres) to an average of 36.1% (142 acres) in the future, due largely to declining streamflow during the growing season in the long run. Such a reduction in irrigated land would result in an annualized income loss of approximately $29.50 acre^−1^ or $11,636 per farm.

Third, the shadow value of a water right's priority is dictated primarily by the probability distribution of daily streamflow, which determines the marginal change in curtailment probability for an extra improved priority (e.g., making a water right 10 years more senior). This contrasts to expected net revenue, which depends on the total volume of streamflow during the growing season. This finding reflects a complex relationship between a water right's priority and its shadow value, and has important implications for water trading. Priority is by no means the sole factor in determining the value of a water right. Rather, a farmer should consider the extent to which a marginal improvement in water right's priority would reduce the curtailment probability relative to their existing curtailment situation.

One final broad insight, relevant to future farm‐level studies, is that the presence of an institutional arrangement on water sharing clearly modulates the effects of water supply uncertainty on farmers' irrigated land use decision and income. Therefore, factors of water institution should be incorporated into farm‐level models (e.g., a streamflow‐curtailment probability curve), along with greater hydrologic details (e.g., skewness in a water supply's probability distribution).

## Supporting information

Supporting Information S1

## Appendix A

### A1. Proof of Proposition 1

To determine the sign of *dL^*^*/*dV*, we must determine the signs of the denominator and numerator on the right‐hand side (RHS) of equation (6). It is easy to show *E*(*U*″Φ^2^) < 0. The signs of remaining terms, however, are less straightforward. Taylor series approximation is then applied to separate the random terms from the deterministic terms. We only consider the Taylor series approximation up to the second order throughout the following discussion in order to be simple and consistent.

Taking the first‐order Taylor approximation of *y*(*w*) and *y*′(*w*) around $\bar{w}$ yields

(A1) $$y\left(w\right)≅y_{0}+y_{0}^{\prime }hɛ,$$

(A2) $$y\prime \left(w\right)≅y_{0}^{\prime }+y_{0}^{\prime \prime }hɛ,$$

where variables with the subscript 0 are *deterministic* terms evaluated at $\bar{w}$. Let *y*″(*w*) ≅ *y*_0_″ and replacing *y*″ with *y*_0_″ in equation (5) yields

(A3) $$\Phi_{L}≅y_{0}^{\prime \prime }\frac{{\left(\bar{w}+ɛh\right)}^{2}}{L},$$

which implies that

(A4) $$E\left(U^{\prime }\Phi_{L}\right)≅\frac{y_{0}^{\prime \prime }}{L}E\left(U^{\prime }{\left(\bar{w}+ɛh\right)}^{2}\right)<0.$$

Therefore, the denominator of the RHS term of equation (6) is negative (note that this denominator is exactly the second order condition for maxima). *dL^*^*/*dV* > 0 if the numerator of the RHS term of (6) is positive and *dL^*^*/*dV* < 0 vice versa.

To explore the sign of the numerator, first we approximate *U*′(*π*) and *U*″(*π*) around *π*_0_ (by definition, *π*_0_ is the *deterministic* term of *π* evaluated at $\bar{w}$ such that *π*_0_≡*Ly*_0_ + *R*( $\bar{L}$ − *L*)),

(A5) $$U\prime \left(\pi \right)≅U_{0}^{\prime }+U_{0}^{\prime \prime }y_{0}^{\prime }Hɛ,$$

(A6) $$U\prime \prime \left(\pi \right)≅U_{0}^{\prime \prime }.$$

Then rewriting *π* as *π* ≅ *π*_0_ + *y*_0_′*Hɛ* and differentiating *π* w.r.t. *L* yields

(A7) $$\Phi ≅\Phi_{0}-y_{0}^{\prime \prime }\bar{w}hɛ,$$

where Φ_0_≡*y*_0_ − *R* − *y*_0_′ $\bar{w}$.

The FOC of *E* (*U*′Φ) = 0 implies Φ_0_ > 0 and *E*(Φ) > 0 (Proof: Rearranging FOC gives Φ_0_=[*y*_0_″ $\bar{w}$ *hE* (*U*′*ɛ*)]/*E*(*U*′). The denominator is positive and the sign of Φ_0_ will be determined by the sign of *E* (*U*′*ɛ*). Since *U*″< 0, it must hold that *U*′(*ɛ*)<*U*′(*ɛ* = 0) for *ɛ* > 0 and *U*′(*ɛ*) > *U*′(*ɛ* = 0) for *ɛ* < 0. Multiplying the two inequalities by *ɛ* produces *U*′(*ɛ*)*ɛ* < *U*′(*ɛ* = 0)*ɛ*. Taking expectations of both sides obtains *E*(*U*′*ɛ*) ≤ *U*′(*ɛ* = 0)*E*(*ɛ*) = 0. Thus, Φ_0_ > 0).

Combining equations (A5)–(A7) gives

(A8) $$E\left(U^{\prime \prime }y^{\prime }H^{\prime }\Phi ɛ+U^{\prime }\Phi_{V}\right)≅U_{0}^{\prime \prime }y_{0}^{\prime \prime }H^{\prime }h\sigma^{2}\left(y_{0}-R-3y_{0}^{\prime }\bar{w}-y_{0}^{\prime \prime }\bar{w}h\sigma \gamma \right),$$

By definition, *U*_0_″, *y*_0_″, and *H*′ are negative. Therefore, the numerator of the RHS term of (6) will be positive iff $\gamma <\frac{y_{0}-R-3y_{0}^{\prime }\bar{w}}{y_{0}^{\prime \prime }\bar{w}h\sigma }$.

### A2. Proof of Proposition 2

The previous proof has demonstrated that the denominator of the RHS term of equation (8) is negative. *dL^*^*/*dR* < 0 if the numerator of the RHS term of (8) is negative and *dL^*^*/*dR* > 0 vice versa. It is easy to show *E*(*U*′)>0. Replacing *U*″ and Φ with equations (A6) and (A7) gives

(A9) $$E\left(U^{\prime \prime }\Phi \left(\bar{L}-L\right)\right)≅U_{0}^{\prime \prime }\left(\bar{L}-L\right)E\left(\Phi \right)<0.$$

#### A2.1. Calculation of *λ*

Differencing *U^*^* w.r.t. *V* yields

(A10) $$\frac{\partial U^{*}}{\partial V}=H^{\prime }E\left(U^{\prime }y^{\prime }ɛ\right)+H^{\prime }\frac{dL^{*}}{dH}E\left(U^{\prime }\Phi \right).$$

The FOC (4) implies *E*(*U*′Φ) = 0, which gives formula (10).

By definition,

(A11) $$\frac{d\lambda }{d\left|\gamma \right|}=-\frac{d\lambda }{d\gamma }=H^{\prime }HhU_{0}^{\prime }y_{0}^{\prime }y_{0}^{\prime \prime }\sigma^{3}>0.$$

#### A3. Proof of Proposition 3

By definition, *s*′< 0, 0< $F_{S}\left[s\left(V\right)\right]$<1, *U*′> 0, and *U*″Φ^2^+*U*′Φ_*L*_<0. These inequalities imply that *dL^*^*/*dV*>0 iff Φ( $\bar{w}$)> 0.

Re‐arranging equation (14) gives

(A12) $$\Phi \left(0\right)=-\frac{\left(1-F_{S}\left[s\left(V\right)\right]\right)U^{\prime }\left[\pi \left(\bar{w}\right)\right]}{F_{S}\left[s\left(V\right)\right]U^{\prime }\left[\pi \left(0\right)\right]}\Phi \left(\bar{w}\right).$$

Because Φ_*w*_ = −*y*″*w*>0 and $\bar{w}$>0, Φ( $\bar{w}$) > Φ(0). Inserting equation (A12) into the inequality Φ( $\bar{w}$) > Φ(0) and rearranging the new inequality yields

(A13) $$\left\{1+\frac{\left(1-F_{S}\left[s\left(V\right)\right]\right)U^{\prime }\left[\pi \left(\bar{w}\right)\right]}{F_{S}\left[s\left(V\right)\right]U^{\prime }\left[\pi \left(0\right)\right]}\right\}\Phi \left(\bar{w}\right)>0.$$

It is easy to show that the coefficient on Φ( $\bar{w}$) in (A13) is positive. Dividing the coefficient on both sides of (A13) gives Φ( $\bar{w}$) > 0—the necessary and sufficient condition for *dL^*^*/*dV* > 0.

Furthermore, differentiating both sides of equation (15), respectively, w.r.t. |*s′*| and $F_{S}\left[s\left(V\right)\right]$ yields

(A14) $$\frac{d}{d\left|s^{\prime }\right|}\left(\frac{dL^{*}}{dV}\right)=-\frac{d}{ds^{\prime }}\left(\frac{dL^{*}}{dV}\right)=\frac{1}{F_{S}\left[s\left(V\right)\right]-1}{\left(\frac{U^{\prime }\Phi }{U^{\prime \prime }\Phi^{2}+U^{\prime }\Phi_{L}}\right)|}_{w=\bar{w}}>0$$

and

(A15) $$\frac{d}{dF_{S}\left[s\left(V\right)\right]}\left(\frac{dL^{*}}{dV}\right)=\frac{s^{\prime }}{{\left(1-F_{S}\left[s\left(V\right)\right]\right)}^{2}}{\left(\frac{U^{\prime }\Phi }{U^{\prime \prime }\Phi^{2}+U^{\prime }\Phi_{L}}\right)|}_{w=\bar{w}}>0.$$

#### A3.1. Proof of Corollary 2

Differentiating both sides of equation (16), respectively, w.r.t. |*s′*| and $f_{S}\left[s\left(V\right)\right]$ yields

(A16) $$\frac{\partial \lambda }{\partial \left|s^{\prime }\right|}=-\frac{\partial \lambda }{\partial s^{\prime }}=f_{S}\left[s\left(V\right)\right]\left(U_{|w=\bar{w}}^{*}-U_{|w=0}^{*}\right)>0$$

and

(A17) $$\frac{\partial \lambda }{\partial f_{S}\left[s\left(V\right)\right]}=-s^{\prime }\left(U_{|w=\bar{w}}^{*}-U_{|w=0}^{*}\right)>0.$$
