# Supplementary material for: Irrigation, risk aversion, and water right priority under water supply uncertainty
Source: Water Resour Res. 2017 Sep 14;53(9):7885–903. doi: 10.1002/2016WR019779 (PMC5698760; doi:10.1002/2016WR019779)
Supplement: Supplementary file 1 — Supporting Information S1 [file WRCR-53-7885-s001.docx]

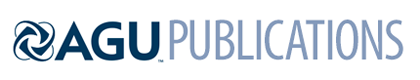


*Water Resources Research*

Supporting Information for

**Irrigation, Risk Aversion, and Water Rights under Water Supply Uncertainty**

Man Li^1^, Wenchao Xu^2^, Mark W. Rosegrant^1^

^1^ Environment and Production Technology Division, International Food Policy Research Institute, Washington, D.C., USA, ^2^ Department of Economics, School of Economics & WISE, Xiamen University, Xiamen, China

**Contents of this file**

Texts S1 to S5

Figures S1 to S5c

**Introduction**

The supplementary materials consist of two portions. The first portion contains four subsections of texts, addressing the farm profit margin calculation, the estimation of the curtailment function, the VIC hydrological model projections under various climate scenarios, and the use of the VIC model data in our simulation studies. We provided the information on the relevant data, the data integration processes, and corresponding issues, if any, related to the processes. The second portion contains five sets of figures. We used the figures to demonstrate the prediction accuracy of the production function and the probability of curtailment, the VIC streamflow projection under CnrmCM3 and HadCM for the Heise Site (A1B and B1) for the year 2009‒2098, the VIC streamflow projection under the HadCM-A1B for the Heise Site for selected years, and a representative farm’s irrigation characteristics under the CnrmCM3-B1, HadCM-A1B, and HadCM-B1 scenarios.

Text S1.

**Farm profit margin calculation**

Agricultural production cost accounts for a large proportion of the total revenue. To calculate the farm profit margin, we extracted Idaho’s county-level crop revenue and farm production expenses from the most recent U.S. Census of Agriculture conducted in the year 2012. Among all production expenses, we considered eight types of expenses that are directly related to crop production, including fertilizer, chemicals, seeds, gasoline and fuels, utilities, repairs/supplies/maintenance, hired farm labor, and rent and lease expenses for machinery/equipment/farm share of vehicles. The expense table reported through the Census of Agriculture does not separate costs related to irrigation activities from other non-irrigation activities. Considering that irrigation is dominant in the state, we assume that irrigation costs have been implicitly included in the expense table. With a pre-assumed share of 24.5% [Painter *et al*. 2010], irrigation expenses were subsequently deducted from the total crop production-related expenses to calculate the production cost for drought-tolerant crops. We further subtracted these expenses from the total revenue, and assigned the county-specific, calculated profit and price margins to each farm based on their respective locations. On average, at the farm level, production cost accounts for 71.2% of total revenue for irrigated crops and accounts for 58.0% of total revenue for drought-tolerant crops.

Text S2.

**Estimating the curtailment function**

We collected the daily natural flow *S* (in cfs, recorded every week) and the corresponding last right information of curtailment (*V*)―the lowest priority level to receive water―for the Heise site (reaches #3 and #4) from April 1 through September 30 during the period of 2009‒2011. For the streamflow cutoff level by which standard the most junior water rights holders (i.e., priority date is in 2009) were eligible to divert water, we only considered the lowest flow level (S = 20,530 cfs) and removed the streamflow above 20,530 cfs from the analysis. This reduced our sample size from 84 observations to 62. We regressed ln(*S*) on *V* with various polynomial specifications from linear to cubic. Overall, both the quadratic and the cubic polynomial specifications fit well (*R*-square ≈ 0.95) and the coefficient estimates under both specifications are statistically significant at the 5% level or better. The cubic specification can capture the structural features of the most junior and the most senior holders in the water rights accounting, but an inverse relationship of *V* and *s*(*V*) is guaranteed by the cubic form. Thus, we chose the piecewise functional form in expression (17) in the manuscript to better represent the real world (Figure S2).

Text S3.

**The VIC streamflow projections under different hydroclimate risk scenarios**

The Columbia Basin Climate Change Scenarios Project (CBCCSP) was conceived as a comprehensive hydrologic database to support climate change planning, impact assessment, and adaptation in the Pacific Northwest. The CBCCSP implemented the Variable Infiltration Capacity (VIC) hydrologic model over the Columbia River Basin to produce two sets of data: the historical simulations (1915‒2006) associated with weather station data and the hydrologic projections (1951‒2098) associated with various general circulation models (GCMs). Figures S3a and S3b demonstrate the hydrologic projections on streamflow from 2009‒2098 under the A1B and B1 emissions scenarios forecasted by two GCMs of the CnrmCM3 and HadCM.

Text S4.

**Calibrating the VIC streamflow in the simulation**

We took multiple steps in the calibration and simulation practice.

First, we constructed a variant of baseline streamflow by taking the average of baseline streamflow on the same date across the three years 2009‒2011. This yields a *28-day baseline* streamflow, where 28 equals the total number of dates when streamflow was recorded from April 1 through September 30 per annum. This 28-day baseline streamflow is invariant to different GCM emission scenarios.^[[1]](#footnote-1)^

Second, we calculated the rate of change in VIC streamflow from its model-specific benchmark level, denoted as *GCM benchmark*. The GCM benchmark was calculated by taking the mean of daily streamflow under each GCM-emission scenario on the same date across 2009‒2011, the same time window as in the baseline scenario. We used this benchmark to adjust the systemic bias of the VIC simulation from the streamflow observations.

Third, we multiplied the 28-day baseline streamflow (obtained from step one) by the rate of change in the VIC streamflow (obtained from step two) under each GCM-emission scenario. The multiplication gives the calibrated VIC streamflow for the period of 2017 through 2098.

The last step of calibration involves the adjustment of the deviation in curtailment probability resulting from taking the three-year average of the baseline streamflow. Specifically, given any water right priority V, we calculated the curtailment probability for each year using the calibrated streamflow under each future GCM-emission scenario. The curtailment probability was then multiplied by an adjustment factor, which equals the division of the baseline curtailment probability (evaluated using the actual 84-day streamflow) by the curtailment probability evaluated using the 28-day baseline streamflow. We used the adjusted curtailment probability to calculate the proportion of land irrigated, the expected net revenue, and the shadow value for the future.

Text S5.

**Evaluating variables in pecuniary terms using the same standard for the baseline versus GCM scenarios**

We treat the 2009‒2011 of the baseline as a single period without adjusting time values within the three years, due to the short time span. Under the GCM scenarios, we treated each year as an individual period, calculating the curtailment probability for each year. We discount the future value of pecuniary variables, because money has interest-earning potential.

Let $g\left( n \right)=\sum_{t=1}^{n} \left( 1+.05 \right)^{-t}=\frac{1-\left( 1+.05 \right)^{-n}}{.05}$, representing a factor of converting a series of constant return over n years to a one-time immediate return, assuming a discount rate of 5% per annum. Mathematically, it equals the summation of the present value factor (1+.05)^-t^ over the whole period.

We report the expected net revenue in annualized value (*Π*) using formula (S1):

$\Pi=\left\{ \begin{aligned} \pi_{0} , baseline scenario \\ \frac{\sum_{t=1}^{82} \pi_{t}\left( 1+.05 \right)^{-t}}{g\left( 82 \right)}, &GCM emission scenario \end{aligned} \right.$, (S1)

where *π_t_* represents the expected net revenue at time *t* (*t* = 0, 1, 2, …, 82); *t* = 0 representing the baseline (status quo) case and t > 0 representing alternative GCM emission scenarios. Note that value of *π_t_* changes over time when *t* > 0. Formula (1) allows us to convert the original, uneven *π_t_* to a constant measure *Π*, which corresponds to π0 under the baseline. Such a conversion is typical in business and economics.

The shadow value of water right priority is measured in present lump-sum value (*Λ*) over an infinite period, as shown in formula (S2):

$\Lambda=\left\{ \begin{aligned} \lambda_{0}g\left( \infty\right) , baseline scenario \\ \left[ \sum_{t=1}^{82} \lambda_{t}\left( 1+.05 \right)^{-t} \right]\frac{g\left( \infty\right)}{g\left( 82 \right)}, &GCM emission scenario \end{aligned} \right.$, (S2)

where *λ_t_* represents the marginal benefit from a ten-year improvement in priority date at time t (t = 0, 1, 2, …, 82). Because water rights are real properties, their values shall reflect the total present value of all *λ_t_* over an infinite period. We assume that *λ_0_* remains unchanged over time under the baseline. For the GCM-emission scenarios, we first calculated the total present value of *λ_t_* over the period of 2017 through 2098, which equals $\sum_{t=1}^{82} \lambda_{t}\left( 1+.05 \right)^{-t}$. Then we used the factor $\frac{g\left( \infty\right)}{g\left( 82 \right)}$ to extend the 82 years to an infinite period.


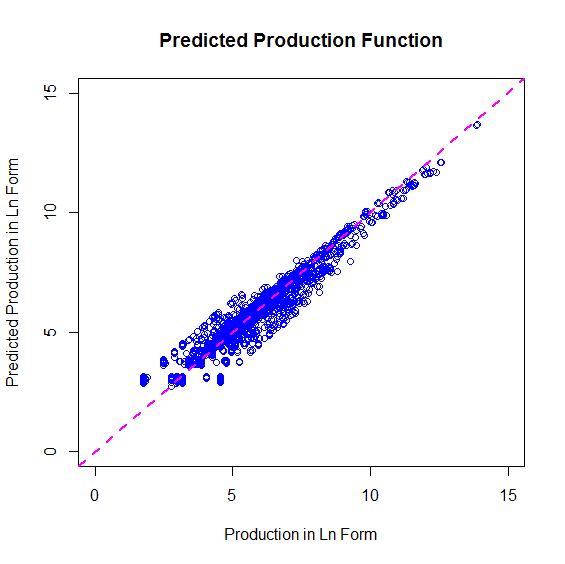


Figure S1. Prediction accuracy of the production function of water-intensive crops

Source: Authors.

Figure S2. Observed vs. predicted probability of curtailment

Source: Authors.


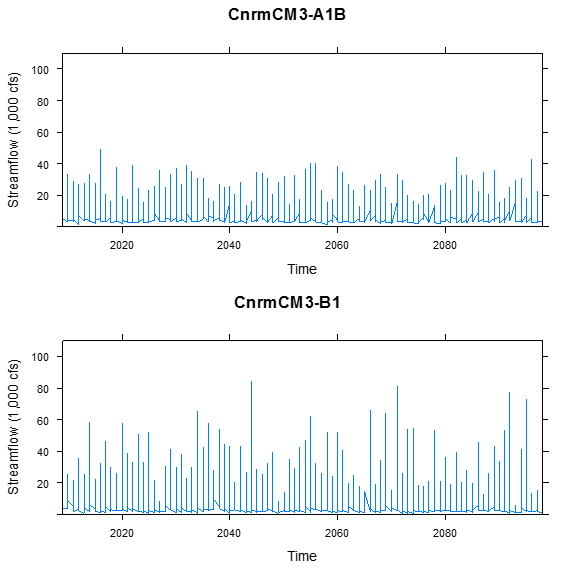


Figure S3a. The VIC streamflow projection forecasted by the CnrmCM3 for the Heise Site, 2009‒2098

Source: Authors’ calculation based on IDWR [2009‒2011] and Hamlet et al. [2010].


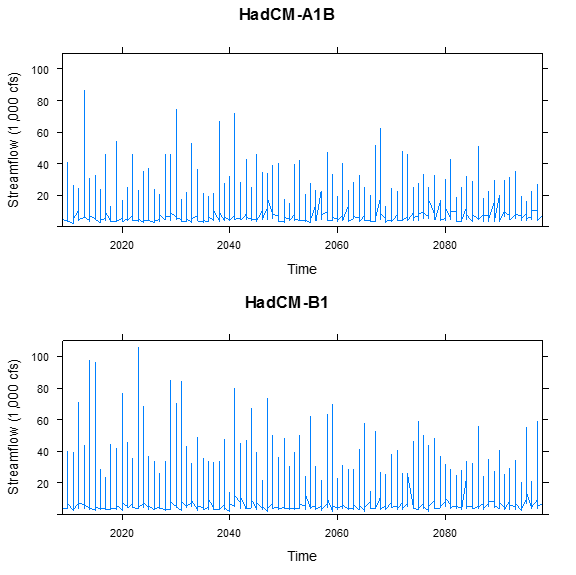


Figure S3b. The VIC streamflow projection forecasted by the HadCM for the Heise Site, 2009‒2098

Source: Authors’ calculation based on IDWR [2009‒2011] and Hamlet et al. [2010].

Figure S4. The VIC streamflow projection under the HadCM-A1Bfor the Heise Site for selected years

Source: Authors’ calculation based on IDWR [2009‒2011] and Hamlet et al. [2010].


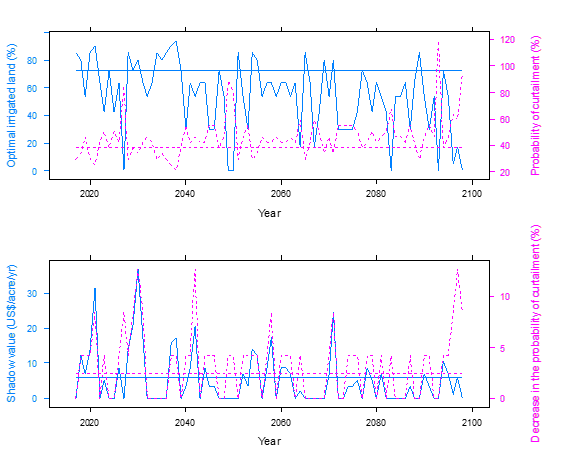


Figure S5a. A representative farm’s irrigation characteristics under the CnrmCM3-B1 scenario (*V* = 0.674)

Source: Authors.

*Note*: The horizontal lines represent the corresponding baseline level. The shadow value presented here is an *undiscounted* value on an annual basis.


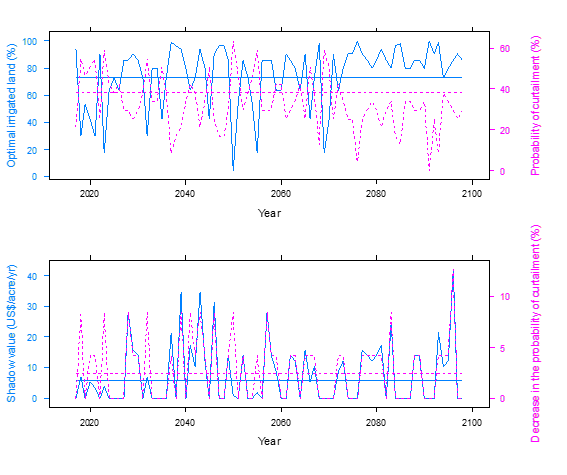


Figure S5b. A representative farm’s irrigation characteristics under the HadCM-A1B scenario (*V* = 0.674)

Source: Authors.

*Note*: The horizontal lines represent the corresponding baseline level. The shadow value presented here is an *undiscounted* value on an annual basis.


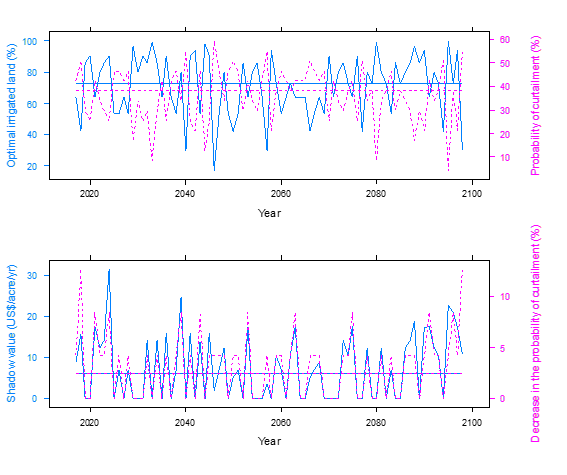


Figure S5c. A representative farm’s irrigation characteristics under the HadCM-B1 scenario (*V* = 0.674)

Source: Authors.

*Note*: The horizontal lines represent the corresponding baseline level. The shadow value presented here is an *undiscounted* value on an annual basis.

1. Note that the baseline results were generated by using the actual 84-day streamflow from 2009 through 2011. The 28-day baseline streamflow calculated here was only used to calibrate the VIC streamflow projected under the GCM-emission scenarios. Because curtailment probability is nonlinear in daily streamflow, simply evaluating the probability at an average of daily streamflow across the three years would bias the baseline results. [↑](#footnote-ref-1)
